# Trends in sperm quality by computer-assisted sperm analysis of 49,189 men during 2015–2021 in a fertility center from China

**DOI:** 10.3389/fendo.2023.1194455

**Published:** 2023-07-12

**Authors:** Yanquan Li, Tingting Lu, Zhengmu Wu, Zhengquan Wang, Ting Yu, Hanshu Wang, Chunhua Tang, Yuchuan Zhou

**Affiliations:** ^1^ International Peace Maternity and Child Health Hospital, School of Medicine, Shanghai Jiao Tong University, Shanghai, China; ^2^ Shanghai Key Laboratory of Embryo Original Diseases, Shanghai, China

**Keywords:** sperm quality, temporal trends, computer-assisted sperm analysis, kinematic parameters, multiple linear regression model

## Abstract

**Background:**

Sperm quality, including semen volume, sperm count, concentration, and total and progressive motility (collectively, “semen parameters”), has declined in the recent decades. Computer-assisted sperm analysis (CASA) provides sperm kinematic parameters, and the temporal trends of which remain unclear. Our objective is to examine the temporal trend of both semen parameters and kinematic parameters in Shanghai, China, in the recent years.

**Methods:**

This retrospective study analyzed semen parameters and kinematic parameters of 49,819 men attending our reproductive center by using CASA during 2015–2021. The total sample was divided into two groups: samples that surpassed the WHO guideline (2010) low reference limits (“above reference limit” group, ARL; n = 24,575) and samples that did not (“below reference limit” group, BRL; n = 24,614). One-way analysis of variance, Kruskal–Wallis test, independent samples *t*-test, and covariance analysis were used to assess the differences among groups. Year, age, and abstinence time were included in the multiple linear regression model of the ARL group to adjust the confounders and depict the trends in sperm quality.

**Results:**

Among all the total sample and the ARL and BRL groups, the age of subjects increased in recent years. Semen volume and sperm count showed declined tendency with years in the total sample, the ARL and BRL groups, and the subgroup of age or abstinence time, whereas sperm velocities showed increased tendency with years on the contrary. The multiple linear regression model of the ARL group, adjusting for age and abstinence time, confirmed these trends. Semen volume (β1= −0.162; CI: −0.172, −0.152), sperm count (β1= −9.97; CI: −10.813, −9.128), sperm concentration (β1 = −0.535; CI: −0.772, −0.299), motility (β1 = −1.751; CI: −1.830, −1.672), and progressive motility (β1 = −1.12; CI: −0.201, −0.145) decreased with year, whereas curvilinear line velocity (VCL) (β1 = 3.058; CI: 2.912, 3.203), straight line velocity (VSL) (β1 = 2.075; CI: 1.990, 2.161), and average path velocity (VAP) (β1 = 2.305; CI: 2.224, 2.386) increased over time (all *p* < 0.001). In addition, VCL, VSL, and VAP significantly declined with age and abstinence time.

**Conclusion:**

The semen parameters declined, whereas the kinematic parameters increased over the recent years. We propose that, although sperm count and motility declined over time, sperm motion velocity increased, suggesting a possible compensatory mechanism of male fertility.

## Introduction

Semen quality has been drawing increasing concerns in the recent decades as the fertility rate has declined and infertility problems have become cumulatively serious (1, 2). In the late 20th century, sperm concentration and semen volume received the most attention after a systematic review reporting that the two parameters had declined by about 50% over the 50 years from 1930 to 1991 (3). Since then, reports from various regions demonstrated the sperm quality–deteriorated trend including decreased sperm count, concentration, normal morphology rate, and total and progressive motility rates (4–13). In China, scholars held a similar view, as reported in studies from Hunan (14), Shandong (15), Henan (16), and other regions (17, 18). Recently, a systematic review and meta-regression analysis revealed that sperm concentration and total sperm count declined worldwide between 1973 and 2018, and the decline in the 21st century was more rapid than that in the last century (19). However, the decline remains controversial as some studies reported no significant change or even an increase in these parameters (20–23). Evidently, male fertility decline is destined to be a crucial long-term issue.

Computer-assisted sperm analysis (CASA) is a notable technological advancement that has gradually replaced manual sperm assessment in reproductive centers worldwide. The advantages of CASA lie in its rapid and automatic semen analysis, providing sample statistics and sperm kinematic parameters including curvilinear line velocity (VCL, μm/s), straight line velocity (VSL, μm/s), average path velocity (VAP, μm/s), straightness (STR, %), linearity (LIN, %), the amplitude of lateral head displacement (ALH, μm), beat cross frequency (BCF, Hz), and wobble (WOB, %) (24). These parameters were difficult to acquire by manual analysis. Only a few studies have reported on the clinical implications or trends alterations associated with these parameters (25–28) especially temporal trend of kinematic parameters. The conclusions drawn from CASA parameters or the significance of these parameters need to be evaluated by more investigations and researches.

Sperm count and motility rate as well as kinematic status are pivotal for fertilization. Activated and hyperactivated sperm display specific movement patterns, characterized by high VSL or high VCL and ALH, aimed to propel sperm migration from the cervix to the oviduct and to enhance cumulus cell layers and zona pellucida penetration during fertilization (29–33). VCL and STR are important references for choosing the therapeutic regimen in assisted reproductive technology. *In vitro* fertilization (IVF) should be considered instead of intracytoplasmic sperm injection if VCL > 65 μm/s and STR > 40 μm/s (34, 35). VSL, VCL, and VAP are valuable in predicting the fertilization potential of spermatozoa in IVF (36–38), whereas STR and BCF can help predict sperm DNA damage (39). The clinical significance of sperm kinematic parameters for fertilization remains open.

This study explored changes in sperm count, motility, and kinematic parameters in the recent years, analyzing these parameters by year, age, and abstinence time. A comprehensive sperm quality evaluation could provide new ideas on elucidating unexplained male infertility and suggest reference values for the kinematic parameters.

## Materials and methods

### Study population and semen samples

We retrieved data on 83,708 samples assessed at the reproductive center of the International Peace Maternity and Child Health Hospital, Shanghai, China, between January 2015 and July 2021. Subsequently, the samples were screened by the following participants and sample characteristics (1): age, 18–60 (2); abstinence time, 2–7 days; and (3) properly formatted and complete information. Only the first sample report was used when more than one sample from the same individual was available. Finally, 49,189 samples were retained. Although the justifications for sperm detection were not specifically analyzed, most analyses were performed in conjunction with pre-pregnancy clinic and infertility clinic including infertility workup of the patient and his partner, as well as oligo-atheno-spermia and sperm freezing for Assisted Reproductive Technology (ART) cycles. Nearly half of the latter category would have subfertility in the male partner. Because of this, the study population would have been biased toward subfertility and not a random cross section of men of reproductive age.

### Ethical approval

This study was under the approval of the Ethics Committee on human subjects of International Peace Maternity and Child Health Hospital (GKLW2018-03).

### Semen analysis

The semen samples were collected by masturbation and liquefied at 37°C for at least 30 min. Two well-trained technicians performed all diagnostic semen analyses, and the results were verified by an andrology-trained laboratory director. Sperm volume was evaluated by reading the values directly from the graduated container. Other semen and kinematics parameters were assessed by CASA (Hamilton-Thorne, Beverly, MA, USA). Each sample (5 μl) was loaded into a counting chamber (Leja Products B.V, Nieuw-Vennep, The Netherlands) and analyzed at once. Ten microscopic fields of each chamber were analyzed, evaluating at least 200 spermatozoa. The following parameters were recorded: total count (million), sperm concentration (million/ml), motility (%), progressive motility (%), VAP (μm/s), VSL (μm/s), VCL (μm/s), STR (%), LIN (%), ALH (μm), BCF (Hz), and WOB (%). Sperm were classified as motile when their path velocity exceeded 5 μm/s. Those sperm with path velocities >25 μm/s and linearity > 80% were classified as “progressively motile sperm.”

The samples were classified as two groups: samples that surpassed the low reference limits (“above reference limit” group, ARL; n = 24,575) and samples that did not (“below reference limit” group, BRL; n = 24,614) according to the World Health Organization laboratory manual for the examination and processing of human semen (fifth edition) (sperm volume, 1.5 ml; sperm count, 39 million; sperm concentration, 15 million/ml; total motility, 40%; and progressive motility, 32%). The samples were also grouped by age (18–24, 25–29, 30–34, 35–39, 40–44, and 45–60 years) and abstinence time (2, 3, 4, 5, 6, and 7 days) to observe trends in these parameters.

### Data analysis

IBM SPSS Statistics for Windows (version 22.0; IBM Corp., Armonk, NY, USA), GraphPad Prism Software (version 8.3; GraphPad Inc., San Diego, CA, USA), and R studio (v.4.1.1; Platform, 64 bit) were used for the statistical analysis and plotting. Semen parameters showed a positively skewed distribution, whereas kinematic parameters were nearly normally distributed. Nonnormally distributed data, semen volume, sperm count, motility, and progressive motility were presented as medians and interquartile range in the tables, and Kruskal–Wallis test was used in the variation analysis, whereas kinematic parameters were shown as means and standard deviation and one-way analysis of variance (ANOVA), independent samples *t*-test, and covariance analysis were used to assess the differences among groups. Means with confidence intervals (95% CIs) were used in line graphs to depict original trend of parameters. A multiple linear regression model was used to explore the changes in the ARL group parameters controlling for two of three factors: year, age, and abstinence time. The R packages “ggpredict” and “ggplot2” were used to visualize the generalized linear model outcomes. The statistical significance is represented as *p* < 0.05, *p* < 0.01, and *p* < 0.001 or non-significance with *p* ≥ 0.05.

## Results

### Distribution of samples

**Figure 1:**
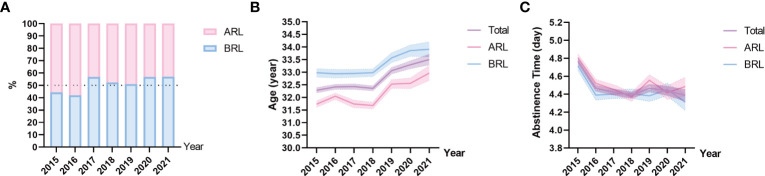
Sample distribution on type, age, and abstinence time. **(A)** Proportions of the above reference limit group (ARL) and the below reference limit group (BRL). **(B, C)** Line charts of age **(B)**, and abstinence time **(C)** based on means with 95% confidence intervals (CIs) over the years. The purple line represents the total sample, the pink line represents the ARL group, and the blue line represents the BRL group.

The dataset includes 49,189 semen samples, divided into ARL (*n* = 24,575, 49.96%) and BRL (*n* = 24,614, 50.04%) groups, and the samples of each year were shown in **Figure 1A**. As shown in **Figures 1B, C** and **Table 1**, the age of the outpatients differed significantly among years in all groups (all *p* < 0.001) and showed an increase toward. Abstinence time had significant difference among years with fluctuation toward. The mean age of participants was 32.59 ± 4.50 years, and the mean abstinence time was 4.50 ± 1.70 days in the total sample.

**Table 1:** Age and abstinence time distribution in the total sample and the ARL and BRL groups over the years.

Year	n	Total sample		n	ARL group		n	BRL group	
		Age (year)	Abstinence time (day)		Age (year)	Abstinence time (day)		Age (year)	Abstinence Time (day)
2015	9,410	32.28 ± 5.09	4.77 ± 1.64	5,240	31.73 ± 4.65	4.81 ± 1.59	4,170	32.98 ± 5.52	4.72 ± 1.69
2016	9,433	32.42 ± 5.11	4.47 ± 1.71	5,467	32.05 ± 4.70	4.52 ± 1.68	3,966	32.94 ± 5.58	4.39 ± 1.75
2017	8,580	32.43 ± 5.20	4.42 ± 1.68	3,710	31.74 ± 4.68	4.45 ± 1.63	4,870	32.95 ± 5.51	4.40 ± 1.72
2018	9,142	32.36 ± 5.02	4.39 ± 1.74	4,361	31.68 ± 4.47	4.37 ± 1.68	4,781	32.98 ± 5.40	4.41 ± 1.79
2019	6,058	33.06 ± 5.15	4.47 ± 1.73	2,965	32.53 ± 4.66	4.56 ± 1.69	3,093	33.57 ± 5.54	4.38 ± 1.76
2020	4,217	33.30 ± 5.20	4.44 ± 1.71	1,822	32.56 ± 4.63	4.42 ± 1.66	2,395	33.86 ± 5.54	4.45 ± 1.74
2021	2,349	33.50 ± 5.25	4.39 ± 1.69	1,010	32.97 ± 4.79	4.49 ± 1.67	1,339	33.91 ± 5.53	4.31 ± 1.71
Total	49,189	32.59 ± 4.50	4.50 ± 1.70	24,575	32.00 ± 4.65	4.54 ± 1.66	24,614	33.18 ± 5.52	4.45 ± 1.74
*p^a^*		< 0.001	< 0.001		< 0.001	< 0.001		< 0.001	< 0.001

Values are presented as mean ± standard deviation. ARL, above reference limit group; BRL, below reference limit group. *p^a^*, One-way analysis of variance (ANOVA) of differences among the 7 years (2015–2021).

### Changes in semen parameters

**Tables 2**, **3** illustrated semen parameters’ medians and interquartile range in the total sample and ARL group over the years. The means and 95% CIs of the parameters in the total sample and ARL and BRL groups were shown in **Figures 2A–E**. These results displayed that semen volume (**Figure 2A**) and sperm count (**Figure 2B**) declined over the years, whereas sperm concentration (**Figure 2C**), motility (**Figure 2D**), and progressive motility (**Figure 2E**) had no significant trends.

**Table 2:** Semen parameters in the total sample during 2015–2021.

Year	n	Semen volume (ml)	Total sperm count (× 10^6^)	Sperm concentration (× 10^6^/ml)	Total motility(%)	Progressive motility (%)
2015	9,410	3.0 (2.0–4.5)	116.96 (57.71–214.06)	38.65 (20.10–64.79)	53.40 (35.93–67.50)	41.96 (26.53–55.43)
2016	9,433	2.8 (2.0–4.0)	116.18 (58.11–206.02)	42.47 (22.66–71.37)	53.20 (37.10–66.10)	44.15 (29.19–56.58)
2017	8,580	2.5 (2.0–4.0)	110.10 (56.67–188.22)	42.21 (24.19–68.89)	41.90 (27.40–54.00)	35.06 (22.16–46.59)
2018	9,142	2.5 (2.0–4.0)	100.68 (51.59–174.02)	38.47 (21.72–63.03)	43.50 (30.10–54.80)	37.50 (25.00–48.13)
2019	6,058	2.5 (2.0–3.1)	87.90 (45.34–159.23)	36.55 (20.70–61.81)	46.10 (32.50–57.60)	39.22 (26.63–50.24)
2020	4,217	2.5 (2.0–3.0)	95.58 (48.67–161.49)	39.06 (21.82–63.32)	42.70 (28.40–54.60)	34.25 (21.97–46.12)
2021	2,349	2.0 (2.0–3.0)	83.75 (44.38–142.27)	37.78 (21.60–63.33)	44.00 (29.35–56.00)	35.68 (22.21–46.64)
*p^a^*		*< 0.001*	*< 0.001*	*< 0.001*	*< 0.001*	*< 0.001*

Values are presented as median (interquartile range).

*p^a^*, The Kruskal–Wallis test of the medians among the years.

**Table 3:** Semen parameters in the ARL group during 2015–2021.

Year	n	Semen volume (ml)	Total sperm count (× 10^6^)	Sperm concentration (× 10^6^/ml)	Total motility (%)	Progressive motility (%)
*2015*	5,240	3.2 (2.5–5.0)	164.68 (101.89–258.52)	48.07 (31.33–73.80)	63.68 (53.89–73.00)	52.19 (42.93–61.16)
*2016*	5,467	3.0 (2.2–4.1)	157.86 (97.33–250.85)	50.86 (32.62–79.28)	61.70 (52.70–71.00)	52.45 (43.59–61.65)
*2017*	3,710	3.0 (2.0–4.0)	152.31 (98.15–230.90)	51.94 (34.62–76.54)	53.60 (47.00–61.40)	46.30 (40.03–53.62)
*2018*	4,361	3.0 (2.0–4.0)	134.51 (86.80–206.47)	47.11 (32.02–69.98)	53.40 (47.00–61.10)	46.57 (40.65–54.42)
*2019*	2,965	3.0 (2.0–3.5)	125.61 (81.08–193.60)	45.67 (29.79–70.14)	55.50 (48.10–63.20)	48.06 (41.08–55.66)
*2020*	1,822	3.0 (2.0–3.5)	132.43 (87.60–197.72)	48.97 (32.64–71.62)	54.65 (47.60–62.20)	46.23 (39.96–53.24)
*2021*	1,010	2.0 (2.0–3.0)	116.89 (78.12–170.56)	48.09 (32.14–72.53)	55.15 (48.30–63.30)	45.96 (40.07–53.95)
*p^a^*		*< 0.001*	*< 0.001*	*< 0.001*	*< 0.001*	*< 0.001*

Values are presented as median (interquartile range).

*p^a^*, The Kruskal–Wallis test of the medians among the years.

**Figure 2:**
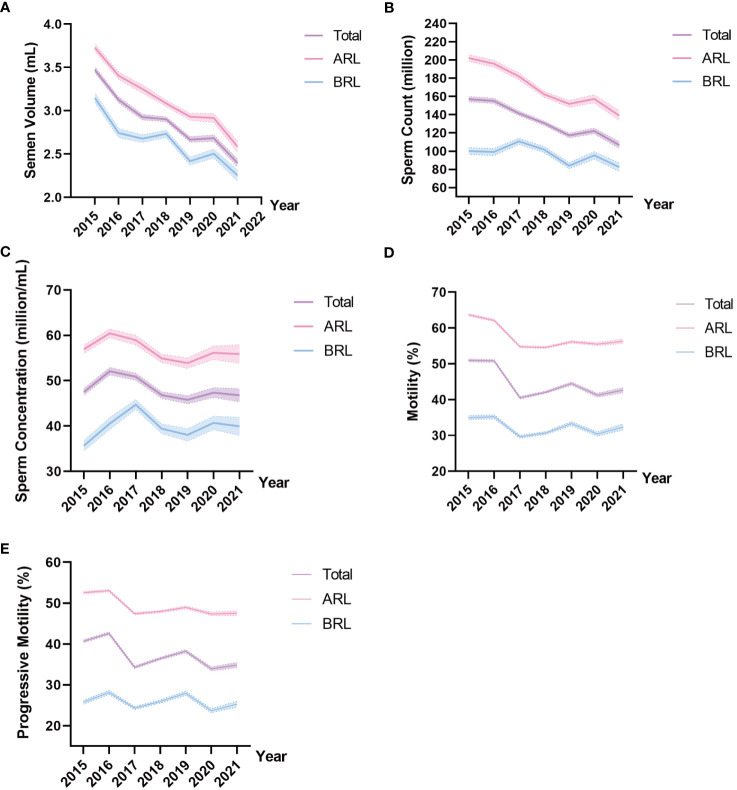
Line charts of semen parameters in the three type groups. Means and 95% CIs of semen volume **(A)**, sperm count **(B)**, sperm concentration **(C)**, motility **(D)**, and progressive motility **(E)** in the groups over the years. The purple line represents the total sample, the pink line represents the ARL group, and the blue line represents the BRL group.

### Changes in sperm kinematic parameters

**Figure 3:**
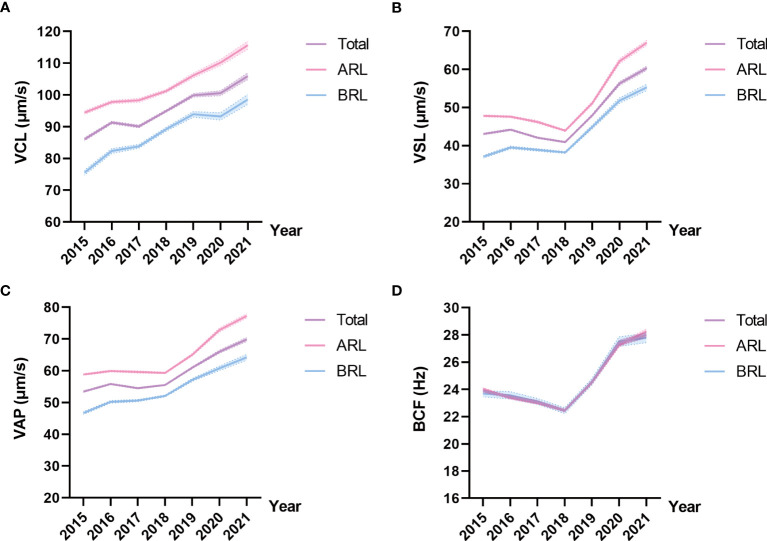
Line charts of kinematic parameters in the three type groups. Means and 95% CIs of VCL **(A)**, VSL **(B)**, VAP **(C)**, and BCF **(D)** in the groups over the years. The purple line represents the total sample, the pink line represents the ARL group, and the blue line represents the BRL group.

Representative kinematic parameters in the total sample and the ARL group sample over the years were shown in **Tables 4**, **5**, respectively. The means and 95% CIs of the parameters in the total sample and the ARL and BRL groups were shown as line graphs in **Figures 3A–D**. The data showed that VCL (**Figure 3A**), VSL(**Figure 3B**), VAP (**Figure 3C**), and BCF (**Figure 3D**) had increased toward with time.

**Table 4:** Sperm kinematic parameters of the total sample during 2015–2021.

Year	n	VCL	VSL	VAP	BCF
2015	9,410	86.05 ± 25.22	43.08 ± 12.76	53.45 ± 13.98	23.88 ± 5.60
2016	9,433	91.31 ± 23.77	44.19 ± 11.94	55.84 ± 12.81	23.45 ± 6.20
2017	8,580	90.09 ± 21.94	42.05 ± 11.62	54.52 ± 12.10	23.07 ± 6.09
2018	9,142	94.94 ± 21.09	40.94 ± 9.37	55.54 ± 10.22	22.45 ± 5.80
2019	6,058	99.88 ± 23.29	47.99 ± 13.98	60.99 ± 13.58	24.53 ± 5.39
2020	4,217	100.55 ± 27.08	56.30 ± 16.91	65.98 ± 17.40	27.40 ± 7.04
2021	2,349	105.93 ± 27.48	60.34 ± 15.05	69.85 ± 16.47	27.99 ± 5.30
*p^a^*		*< 0.001*	*< 0.001*	*< 0.001*	*< 0.001*

Values are presented as mean ± standard deviation.

*p^a^*, The one-way ANOVA of the kinematics parameters among the years.

**Table 5:** Sperm kinematic parameters in the ARL group during 2015–2021.

Year	n	VCL	VSL	VAP	BCF
2015	5,240	94.43 ± 21.55	47.82 ± 10.59	58.84 ± 11.20	24.04 ± 3.42
2016	5,467	97.79 ± 20.60	47.58 ± 10.36	59.92 ± 10.57	23.37 ± 3.33
2017	3,710	98.31 ± 19.10	46.20 ± 10.89	59.63 ± 10.36	23.00 ± 3.29
2018	4,361	101.23 ± 18.40	43.92 ± 8.56	59.34 ± 8.44	22.45 ± 2.79
2019	2,965	106.12 ± 20.37	51.17 ± 13.35	65.05 ± 12.03	24.50 ± 3.80
2020	1,822	110.18 ± 21.33	62.16 ± 14.89	72.83 ± 14.01	27.25 ± 3.64
2021	1,010	115.67 ± 21.28	67.01 ± 10.52	77.29 ± 11.26	28.26 ± 2.97
*p^a^*		*< 0.001*	*< 0.001*	*< 0.001*	*<0.001*

Values are presented as mean ± standard deviation.

*p^a^*, The one-way ANOVA of the kinematics parameters among the years in the ARL group.

The kinematic parameters of the ARL group—VCL, VSL, VAP, ALH, LIN, and STR—were higher than those of the BRL group (all *p* < 0.001; **Table 6**), even after adjusting for age and abstinence time. These parameters could discriminate the sperm motility coincident with semen parameters. Whereas, BCF and WOB of the ARL group were lower than BRL group after the adjustment (*p* < 0.001).

**Table 6:** Variation analysis of kinematic parameters in the different groups.

Parameter	Total sample n = 49,819	ARL n = 24,575	BRL n = 24,614	*p^a^*	*p^b^*
VCL	93.3123 ± 24.3496	100.4208 ± 21.0102	86.2151 ± 25.3642	*<0.001*	*<0.001*
VSL	45.2764 ± 13.6507	49.0876 ± 12.4259	41.4713 ± 13.7570	*<0.001*	*<0.001*
VAP	57.2697 ± 14.0085	61.8333 ± 11.9238	52.7134 ± 14.4454	*<0.001*	*<0.001*
LIN	50.4537 ± 9.3838	50.6666 ± 9.0333	50.2412 ± 9.7166	*<0.001*	*<0.001*
ALH	5.0330 ± 1.3248	5.2976 ± 1.2017	4.7687 ± 1.3878	*<0.001*	*<0.001*
STR	77.5406 ± 8.5594	77.8576 ± 7.8030	77.2240 ± 9.2425	*<0.001*	*<0.001*
BCF	23.9664 ± 6.1385	23.9188 ± 3.6441	24.0139 ± 7.8766	*0.086*	*<0.001*
WOB	63.0401 ± 5.9304	62.9979 ± 5.5934	63.0823 ± 6.2486	*0.114*	*<0.001*

Values are presented as mean ± standard deviation.

*p^a^*, The independent samples t-test of the kinematic parameters between the ARL and BRL groups.

*p^b^*, Covariance analysis of the kinematic parameters between the ARL and BRL groups, adjusted for age and abstinence time.

### Multiple linear regression model of ARL group

**Figure 4:**
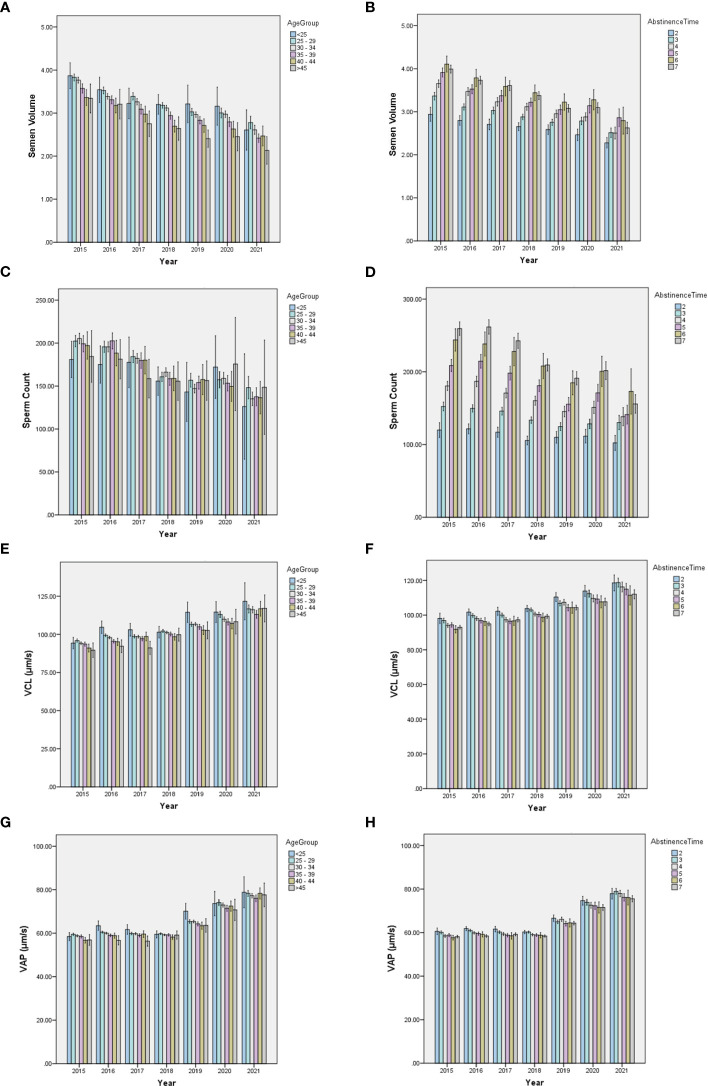
Bar charts of semen quality parameters in subdivided ARL groups by age or abstinence time. The yearly distribution of semen volume, sperm count, VCL, and VAP among the age **(A, C, E, G)** and abstinence time **(B, D, F, H)** groups.

To exclude the potential effects of discrepant age, abstinence time, and possible diseases in participants of the BRL group on temporal trends in sperm quality, we analyzed the ARL group separately by dividing it into subgroups by age or abstinence time (**Figure 4**). The results showed that the individual age (**Figures 4A, C, E, G**) or abstinence time (**Figures 4B, D, F, H**) groups had similar trends over time, although age and abstinence time distributions differed among years. To exhibit the effect of age and abstinence time on sperm quality, line charts of semen and kinematic parameters in the different age and abstinence time groups in the total sample and the ARL and BRL groups were shown in **Figures S1, S2**.

**Figure 5:**
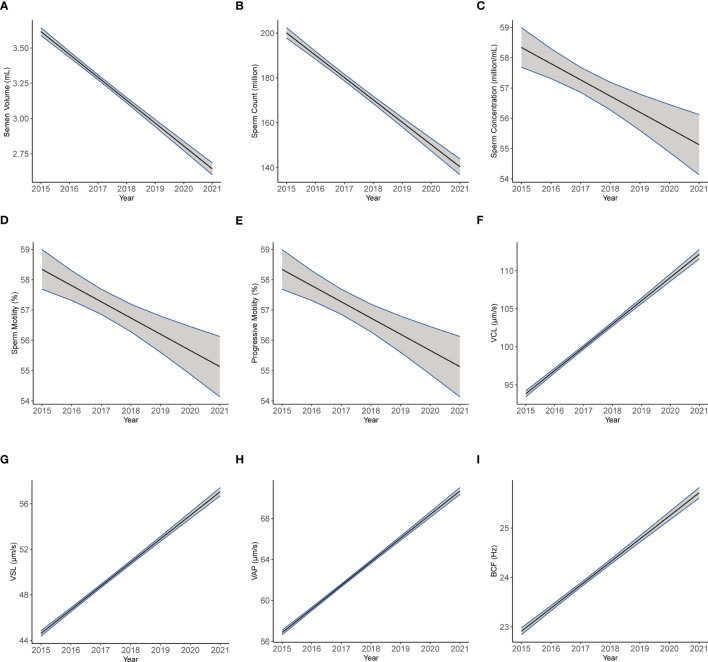
The plotting of multiple regression linear model of semen and kinematics parameters by year. Images of multiple regression linear models fitting of semen parameters: semen volume **(A)**, sperm count **(B)**, sperm concentration **(C)**, motility **(D)**, and progressive motility **(E)** and kinematics parameters: VCL **(F)**, VSL **(G)**, VAP **(H)**, and BCF **(I)** by year adjusted for age and abstinence time.

We established multiple linear regression models to further explore the relationship between semen parameters with year, age, and abstinence time in the ARL group (**Table 7**). After adjusting for age and abstinence time, we found significant declines with year in the semen parameters, including semen volume (β1 = −0.162; CI: −0.172, −0.152; **Figure 5A**), sperm count (β1 = −9.97; CI: −10.813, −9.128; **Figure 5B**), sperm concentration (β1 = −0.535; CI: −0.772, −0.299; **Figure 5C**), total motility (β1 = −1.751; CI: −1.830, −1.672; **Figure 5D**), and progressive motility (β1 = −1.12; CI: −0.201, −0.145; **Figure 5E**). Conversely, we noted significant increases over time in VCL (β1 = 3.058; CI: 2.912, 3.203; **Figure 5F**), VSL (β1 = 2.075; CI: 1.990, 2.161; **Figure 5G**), VAP (β1 = 2.305; CI: 2.224, 2.386; **Figure 5H**), and BCF (β1 = 0.467; CI: 0.441, 0.492; **Figure 5I**; all *p* < 0.001). The estimates should be interpreted as semen volume, sperm count, sperm concentration, motility, and progressive motility decreased: 0.162 ml/year, 9.97 × 10^6^/year, 0.535 × 10^6^/ml/year, 1.751%/year, and 1.12%/year, respectively; whereas, VCL, VSL, VAP, and BCF increased: 3.058 μm/s, 2.075 μm/s, 2.305 μm/s, and 0.467 Hz per year, respectively.

**Table 7:** Multiple regression linear model of semen parameters.

		Semen volume	Sperm count	Sperm concentration	Total motility	Progressive motility	
	Predictors	Estimates (95% CI)	Estimates (95% CI)	Estimates (95% CI)	Estimates (95% CI)	Estimates (95% CI)	*p*
Semen parameters	Year (β1)	−0.162 (−0.172, −0.152)	−9.97 (−10.813, −9.128)	−0.535 (−0.772, −0.299)	−1.751 (−1.830, −1.672)	−1.12 (−1.195, −1.044)	<0.001
	Age (β2)	−0.029 (−0.033, −0.026)	−0.554 (−0.869, −0.239)	0.374 (0.285, 0.462)	−0.139 (−0.168, −0.109)	−0.173 (−0.201, −0.145)	<0.001
	Abstinence Time (β3)	0.150 (0.130, −0.160)	23.033 (22.148, 23.917)	4.666 (4.418, 4.915)	−0.313 (−0.396, −0.229)	−0.527 (−0.60, −0.448)	<0.001
	R^2^	0.080	0.119	0.057	0.077	0.046	
Kinematic parameters		VCL	VSL	VAP	BCF		
	Predictors	Estimates (95% CI)	Estimates (95% CI)	Estimates (95% CI)	Estimates (95% CI)	*p*	
	Year (β1)	3.058 (2.912, 3.203)	2.075 (1.990, 2.161)	2.305 (2.224, 2.386)	0.467 (0.441, 0.492)	<0.001	
	Age (β2)	−0.247 (−0.301, −0.192)	−0.045 (−0.077, −0.013)	−0.091 (−0.122, −0.061)	0.01 (0.001, 0.020)	<0.05	
	Abstinence Time (β3)	−0.967 (−1.120, −0.814)	−0.199 (−0.289, −0.109)	−0.389 (−0.473, −0.304)	0.092 (0.065, 0.118)	<0.001	
	R^2^	0.074	0.086	0.119	0.051		

Multiple linear regression model of sperm analysis parameters. Year, age, and abstinence time were included as factors. β1, β2, and β3 represent the slope of liner regression by year, age, and abstinence time, respectively, weighted by the other two factors. R^2^, R-squared represented the fitness of the model. VCL (p < 0.001), VSL (*p* = 0.006), VAP (*p* < 0.001), and BCF (*p* = 0.035) declined with age.

**Figure 6:**
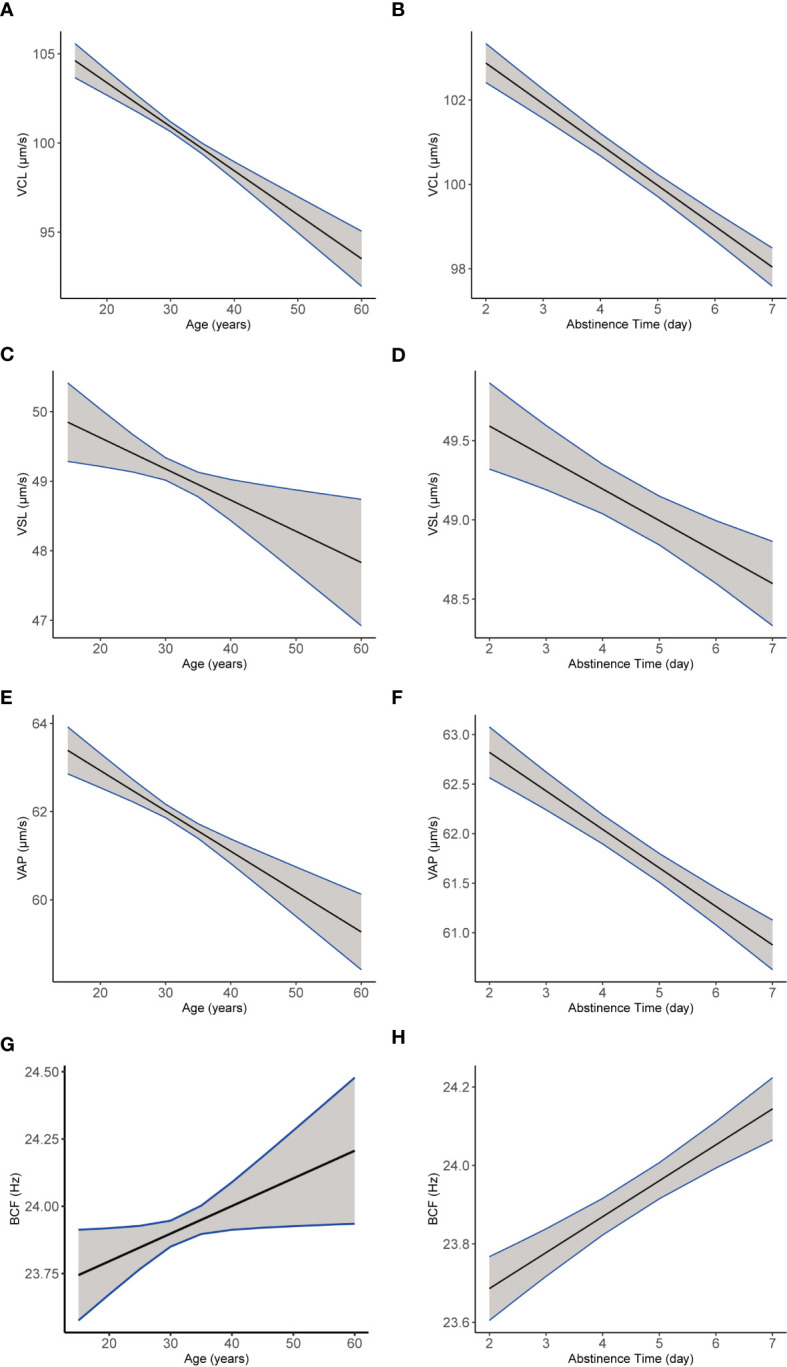
The plotting of multiple regression linear model of semen and kinematics parameters by age or abstinencetime. Images of multiple regression linear models fitting of sperm kinematic parameters VCL **(A)**, VSL **(C)**, VAP **(E)**, and BCF **(G)** by age, adjusted for the year and abstinence time. Images of multiple regression linear models fitting of sperm kinematic parameters VCL **(B)**, VSL **(D)**, VAP **(F)**, and BCF **(H)** by abstinence time (b, d, f, h), adjusted for age and the year.

In addition, most semen parameters declined with age (**Table 7**), including sperm count, semen volume, total motility, and progressive motility, as well as kinematic parameters VCL (**Figure 6A**), VSL (**Figure 6C**), and VAP (**Figure 6E**; all *p* < 0.001). Sperm concentration and BCF (**Figure 6G**, both *p* < 0.001) increased with age. Moreover, abstinence time was associated with increased semen volume, sperm count, sperm concentration, and BCF (**Figure 6H**; all *p* < 0.001). Conversely, total motility, progressive motility, VCL (**Figure 6B**), VSL (**Figure 6D**), and VAP (**Figure 6F**; all *p* < 0.001) declined with abstinence time.

## Discussion

Male sperm quality decline over the years is a worldwide trend. This study found declining trends over the years in semen volume, sperm count, and motility, consistent with most previous studies (4–18). Unexpectedly, a further novel finding is the increasing trend with year in the kinematics parameters VCL, VSL, and VAP, even after correcting for age and abstinence time. In addition, sperm velocity decreased with age and abstinence time as previous studies have reported (25–28). This result suggests that continuous attention and improvement of male fertility remain important scientific issues in the field of reproduction.

The cause and clinical significance of the kinematic parameters’ increase remain unclear. Sperm velocity is inextricably related to the sperm motility state, which is used to discriminate sperm motile state in CASA and increases during capacitation and hyperactivation (40–42). It was also reported that a higher percentage of hyperactivated spermatozoa was related to higher fertilization rates in conventional IVF (40). Our results hinted that sperm movement velocity increased despite a decrease in the amount and rate of motile sperm, which probably compensates to some extent for male fertility. However, we cannot exclude the possibility that the activation of sperm motility might be related to premature hyperactivation, which might cause spontaneous acrosome reaction and unexplained fertilization failure (43). This sequence of events has a detrimental effect on male fertility, especially in men with astheno- and oligozoospermia, possibly another indicator of declining male fertility. However, the data of kinematic parameters are not been reported online enough, which made the verification of our results difficult. We hope that more colleagues will pay attention to these data to discuss the accuracy and significance of this result. The causes and clinical implications of these results also should be explored in future prospective and experimental studies.

The age of subjects included in this study increased between 2015 and 2021. The main reason for this increase may be the introduction of the Chinese fertility policy that eased the one-child policy, causing many older men to visit clinical fertility centers. Moreover, there is a tendency to postpone the fatherhood age with the increase in educational requirements for employment. Therefore, we must consider the distribution of population age when studying the trends in semen parameters over time.

Furthermore, age was a significant factor in sperm quality. Many studies have indicated that age was related to sperm concentration, sperm count, total motility, and progressive motility (28, 44). The kinematic parameters assessed by CASA were also associated with age. Studies showed that ALH, VCL, VSL, VAP, LIN, STR, and head width–to–length ratio significantly decreased with age, suggesting a decline in sperm velocity (25–28). Our results supported this point (**Table 7** and **Supplementary Figure 1**). To exclude the impact of age increase in our study, we compared the semen quality and kinetic parameters of the same age group every year and found that the conclusion that semen quality decreased and sperm motility velocity increased year by year did not change. In addition, age was considered a confounder when analyzing the sperm parameters’ temporal trends in regression models.

Abstinence time can also impact sperm quality (45–47). Sperm count, volume, and concentration increased gradually with abstinence time of up to 10 days (25, 46). Recent studies focusing on clinical assisted reproduction recommended a short ejaculatory abstinence interval to obtain better sperm quality, especially in infertile men (47–52). Our results showed that semen volume, sperm count, and concentration increased, whereas total and progressive motility, VCL, VSL, and VAP decreased with abstinence time (within the range of 2–7 days). The longer the sperm were stored in the epididymis, the poorer its motility was once ejaculated, especially in the BRL group (**Figure S2**). Sperm are continuously stored and naturally renewed in the epididymis. However, many studies suggested that the quality of sperm is closely related to the abstinence time or storage time in the epididymis, especially under pathological or harmful environmental conditions (45, 47). First, previous reports demonstrated that human sperm are susceptible to damage induced by reactive oxygen species (ROS) (53). Increased abstinence periods enhance the ROS-exposed time and bring about lipid peroxidation and malondialdehyde that is harmful to acrosome, mitochondrial activity, and DNA integrity (49, 54–58). For subfertile men, abnormal factors may result in excessive production of ROS in the epididymis, leading to decreased sperm motility and an infertile status (59–61). Impaired antioxidant capacity of abnormal spermatozoa also makes infertile sperm more prone to be damaged by ROS (62–65). Second, longer abstinence period affects secretions of epididymis, prostate, and seminal vesicle, which are related to the pH and biochemical of final semen and affect sperm motility (24, 66, 67). Moreover, longer abstinence time is related to a higher bacterial load and diversity in semen, more pro-inflammatory cytokines (68), as well as decreased rate of morphologically normal sperm (46, 69). Therefore, a shorter abstinence time is suggested for patients with oligo-atheno-zoospermia as it increases the probability of obtaining higher-quality sperm.

The rate of decline in sperm count and sperm concentration derived from this study was slightly higher than previous studies (5, 18, 19) (non-selected population fraction or reproductive center data). This may be related to the demographic and employment characteristics of the city as an economic center. Our research center is located in a mega city of China with high talent competition and socioeconomic burden, and one notable feature that sets it apart from other cities is the high level of work pressure, which, in turn, leads to reduced sleep time, high psychosocial stress, and irregular eating. Studies reported that short sleep durations, late bedtime, and poor sleep quality were associated with impaired sperm health (70, 71). Psychosocial stress was also negatively associated with sperm concentration, total count, and motility (72–74). Unbalanced diet is reported among Shanghai population (75) and is harmful to sperm quality, which includes overconsumption of cooking oil and salt as well as under-intake of dairy, vegetables and fruits, soybeans, fish and shrimps, and eggs (75–79). These lifestyles can easily lead to male nutritional disorders and endocrine dysfunction. For instance, overweight and obesity brought by unhealthy lifestyle are positively associated with high estradiol concentrations and lower sperm quality (80, 81). In addition, as China’s main industrial center, Shanghai has serious air pollution that adversely impacts on sperm quality (82).

The strengths of this study are as follows. Most previous studies reported that semen parameters declined in recent decades, whereas this study focused on sperm kinematic parameters, showing increasing trends of sperm velocity over the years. Furthermore, we used a multiple linear regression model to analyze the data after factoring in the effects of age and abstinence time considering that they may have some impacts on the results. Moreover, systematic error in data was low as the methods, personnel, and instruments were consistent throughout the study period.

However, the study had several limitations. First, the data came from a single reproductive center, and the subjects included healthy and infertile or potentially infertile men, which might not accurately reflect the trends in the general population. Second, we only corrected for age and abstinence time. Other potentially influencing factors were not recorded or analyzed. Third, the alterations in sperm quality do not necessarily indicate changes in fertility, and the fertilization outcomes were unknown. Despite the shortcomings of this research, the analysis of the results is reasonable and reliable. The majority of sperm quality studies are from single-centers with minor instrument and manual operation errors. Statisticians afterward could collect many studies’ data for systematic review and meta-analysis and summarize widely conclusions. As for this study, the amount of sample is large in this study, and this can offset the impact caused by unselected subjects. We have further subdivided the total sample into different groups to diminish the potential interference. In addition, the conclusion that semen quality is deteriorating year by year is in good agreement with previous reports. Thus, the alterations of sperm kinematics parameters based on mutual reports are analogously convincing in this study. Indeed, semen quality is influenced by factors such as occupational factors, living habits, body mass, and environmental adverse factors. It is extremely difficult to eliminate all factors. Thus, it is crucial to overcome these shortcomings for future prospective experimental research design.

The decrease in semen parameters and increase in the kinematic parameters over the years might be the current trend in male fertility. Sperm kinematic parameters reflect information on sperm movement and functional state. The clinical significance of these alterations needs further studies to be verified and explored, which could provide new reference values for guideline formulation and more diagnosis basis for unexplained infertility.

## Conclusion

We presented temporal trends in sperm quality that comprised decreased semen parameters and increased kinematic parameters. Our results implied that enhancive velocity of sperm may be a potential compensatory mechanism of decreased sperm count and motility.

## Data availability statement

The data analyzed in this study is subject to the following licenses/restrictions: The dataset belongs to our institution and data underlying this article will be shared on reasonable request to the corresponding authors. Requests to access these datasets should be directed to zhouych@sibcb.ac.cn.

## Ethics statement

This study was under approval by the Ethics Committee on human subjects of International Peace Maternity and Child Health Hospital (GKLW2018-03). Written informed consent for participation was not required for this study in accordance with the national legislation and the institutional requirements.

## Author contributions

YZ, CT, YL, and TL conceived and designed the study. YL and TL completed most of the data collection and analysis. ZMW, ZQW, HW, and TY participated in the sample collection, data analysis, and further explanation. YZ, CT, YL, and TL wrote the manuscript. All authors contributed to the article and approved the submitted version.

## References

[1] Virtanen HE Jørgensen N Toppari J. Semen quality in the 21(St) century. Nat Rev Urol (2017) 14(2):120–30. doi: 10.1038/nrurol.2016.261 28050014

[2] Agarwal A Baskaran S Parekh N Cho CL Henkel R Vij S. Male Infertility. Lancet (2021) 397(10271):319–33. doi: 10.1016/s0140-6736(20)32667-2 33308486

[3] Carlsen E Giwercman A Keiding N Skakkebaek NE. Evidence for decreasing quality of semen during past 50 years. Bmj (1992) 305(6854):609–13. doi: 10.1136/bmj.305.6854.609 PMC1883354 1393072

[4] Siqueira S Ropelle AC Nascimento JAA Fazano FAT Bahamondes LG Gabiatti JR. Changes in seminal parameters among Brazilian men between 1995 and 2018. Sci Rep (2020) 10(1):6430. doi: 10.1038/s41598-020-63468-9 32286479 PMC7156660

[5] Mínguez-Alarcón L Williams PL Chiu YH Gaskins AJ Nassan FL Dadd R. Secular trends in semen parameters among men attending a fertility center between 2000 and 2017: identifying potential predictors. Environ Int (2018) 121(Pt 2):1297–303. doi: 10.1016/j.envint.2018.10.052 PMC6279498 30389382

[6] Mishra P Negi MPS Srivastava M Singh K Rajender S. Decline in seminal quality in Indian men over the last 37 years. Reprod Biol Endocrinol (2018) 16(1):103. doi: 10.1186/s12958-018-0425-z 30352581 PMC6199708

[7] Levine H Jørgensen N Martino-Andrade A Mendiola J Weksler-Derri D Mindlis I. Temporal trends in sperm count: a systematic review and meta-regression analysis. Hum Reprod Update (2017) 23(6):646–59. doi: 10.1093/humupd/dmx022 PMC6455044 28981654

[8] Sengupta P Nwagha U Dutta S Krajewska-Kulak E Izuka E. Evidence for decreasing sperm count in African population from 1965 to 2015. Afr Health Sci (2017) 17(2):418–27. doi: 10.4314/ahs.v17i2.16 PMC5637027 29062337

[9] Olesen IA Joensen UN Petersen JH Almstrup K Rajpert-De Meyts E Carlsen E. Decrease in semen quality and leydig cell function in infertile men: a longitudinal study. Hum Reprod (2018) 33(11):1963–74. doi: 10.1093/humrep/dey283 30247578

[10] Sugihara A De Neubourg D Punjabi U. Is there a temporal trend in semen quality in Belgian candidate sperm donors and in sperm donors' fertility potential from 1995 onwards? Andrology (2021) 9(3):846–53. doi: 10.1111/andr.12963 33336502

[11] Swan SH Elkin EP Fenster L. The question of declining sperm density revisited: an analysis of 101 studies published 1934-1996. Environ Health Perspect (2000) 108(10):961–6. doi: 10.1289/ehp.00108961 PMC1240129 11049816

[12] Sengupta P Borges E Dutta S Krajewska-Kulak E. Decline in sperm count in European men during the past 50 years. Hum Exp Toxicol (2018) 37(3):247–55. doi: 10.1177/0960327117703690 28413887

[13] Auger J Kunstmann JM Czyglik F Jouannet P. Decline in semen quality among fertile men in Paris during the past 20 years. N Engl J Med (1995) 332(5):281–5. doi: 10.1056/nejm199502023320501 7816062

[14] Huang C Li B Xu K Liu D Hu J Yang Y. Decline in semen quality among 30,636 young Chinese men from 2001 to 2015. Fertil Steril (2017) 107(1):83–8.e2. doi: 10.1016/j.fertnstert.2016.09.035 27793371

[15] Wang L Zhang L Song XH Zhang HB Xu CY Chen ZJ. Decline of semen quality among Chinese sperm bank donors within 7 years (2008-2014). Asian J Androl (2017) 19(5):521–5. doi: 10.4103/1008-682x.179533 PMC5566843 27345004

[16] Liu J Dai Y Li Y Yuan E Wang Q Guan Y. Analysis of the screening results of 24040 potential sperm donors in a human sperm bank in henan province, China: a 14-year retrospective cohort study. Hum Reprod (2021) 36(5):1205–12. doi: 10.1093/humrep/deab028 33611556

[17] Huang L-p Li Y-f Xiong H-y Cao J. Changing tendency analysis of Chinese normal male's semen quality in recent 25 years: samples from Chinese documents. J Reprod Contraception (2010) 21(4):229–41. doi: 10.1016/s1001-7844(11)60005-9

[18] Lv MQ Ge P Zhang J Yang YQ Zhou L Zhou DX. Temporal trends in semen concentration and count among 327 373 Chinese healthy men from 1981 to 2019: a systematic review. Hum Reprod (2021) 36(7):1751–75. doi: 10.1093/humrep/deab124 34046659

[19] Levine H Jørgensen N Martino-Andrade A Mendiola J Weksler-Derri D Jolles M. Temporal trends in sperm count: a systematic review and meta-regression analysis of samples collected globally in the 20th and 21st centuries. Hum Reprod Update (2022) 29(2):157–76. doi: 10.1093/humupd/dmac035 36377604

[20] Swan SH Elkin EP Fenster L. Have sperm densities declined? a reanalysis of global trend data. Environ Health Perspect (1997) 105(11):1228–32. doi: 10.1289/ehp.971051228 PMC1470335 9370524

[21] Li WN Jia MM Peng YQ Ding R Fan LQ Liu G. Semen quality pattern and age threshold: a retrospective cross-sectional study of 71,623 infertile men in China, between 2011 and 2017. Reprod Biol Endocrinol (2019) 17(1):107. doi: 10.1186/s12958-019-0551-2 31815629 PMC6902580

[22] Jørgensen N Joensen UN Jensen TK Jensen MB Almstrup K Olesen IA. Human semen quality in the new millennium: a prospective cross-sectional population-based study of 4867 men. BMJ Open (2012) 2(4):e000990. doi: 10.1136/bmjopen-2012-000990 PMC3391374 22761286

[23] Olsen GW Bodner KM Ramlow JM Ross CE Lipshultz LI. Have sperm counts been reduced 50 percent in 50 years? a statistical model revisited. Fertil Steril (1995) 63(4):887–93. doi: 10.1016/s0015-0282(16)57498-6 7890079

[24] World Health Organization. Who laboratory manual for the examination and processing of human semen. 5th ed Vol. xiv. Geneva: World Health Organization (2010). p. 271.

[25] Chen GX Li HY Lin YH Huang ZQ Huang PY Da LC. The effect of age and abstinence time on semen quality: a retrospective study. Asian J Androl (2022) 24(1):73–7. doi: 10.4103/aja202165 PMC8788608 34747722

[26] Fréour T Jean M Mirallie S Barriere P. Computer-assisted sperm analysis parameters in young fertile sperm donors and relationship with age. Syst Biol Reprod Med (2012) 58(2):102–6. doi: 10.3109/19396368.2011.642054 22175659

[27] Stone BA Alex A Werlin LB Marrs RP. Age thresholds for changes in semen parameters in men. Fertil Steril (2013) 100(4):952–8. doi: 10.1016/j.fertnstert.2013.05.046 23809502

[28] Sloter E Schmid TE Marchetti F Eskenazi B Nath J Wyrobek AJ. Quantitative effects of Male age on sperm motion. Hum Reprod (2006) 21(11):2868–75. doi: 10.1093/humrep/del250 16793993

[29] Suarez SS. Control of hyperactivation in sperm. Hum Reprod Update (2008) 14(6):647–57. doi: 10.1093/humupd/dmn029 18653675

[30] Jin JL Jin NG Zheng HL Ro S Tafolla D Sanders KA. Catsper3 and Catsper4 are essential for sperm hyperactivated motility and Male fertility in the mouse. Biol Reprod (2007) 77(1):37–44. doi: 10.1095/biolreprod.107.060186 17344468

[31] Suarez SS Pacey AA. Sperm transport in the female reproductive tract. Hum Reprod Update (2006) 12(1):23–37. doi: 10.1093/humupd/dmi047 16272225

[32] Qi HY Moran MM Navarro B Chong JA Krapivinsky G Krapivinsky L. All four catsper ion channel proteins are required for Male fertility and sperm cell hyperactivated motility. Proc Natl Acad Sci United States America (2007) 104(4):1219–23. doi: 10.1073/pnas.0610286104 PMC1770895 17227845

[33] Suarez SS Ho HC. Hyperactivated motility in sperm. Reprod Domest Anim (2003) 38(2):119–24. doi: 10.1046/j.1439-0531.2003.00397.x 12654022

[34] Larsen L Scheike T Jensen TK Bonde JP Ernst E Hjollund NH. Computer-assisted semen analysis parameters as predictors for fertility of men from the general population. Danish First Pregnancy Planner Study Team. Hum Reprod (2000) 15(7):1562–7. doi: 10.1093/humrep/15.7.1562 10875866

[35] Dcunha R Hussein RS Ananda H Kumari S Adiga SK Kannan N. Current insights and latest updates in sperm motility and associated applications in assisted reproduction. Reprod Sci (2022) 29(1):7–25. doi: 10.1007/s43032-020-00408-y 33289064 PMC7721202

[36] Joshi N Kodwany G Balaiah D Parikh M Parikh F. The importance of computer-assisted semen analysis and sperm function testing in an ivf program. Int J Fertil Menopausal Stud (1996) 41(1):46–52. 8673156

[37] Donnelly ET Lewis SE McNally JA Thompson W. *In vitro* fertilization and pregnancy rates: the influence of sperm motility and morphology on ivf outcome. Fertil Steril (1998) 70(2):305–14. doi: 10.1016/s0015-0282(98)00146-0 9696226

[38] Liu DY Clarke GN Baker HW. Relationship between sperm motility assessed with the Hamilton-thorn motility analyzer and fertilization rates in vitro. J Androl (1991) 12(4):231–9. doi: 10.1002/j.1939-4640.1991.tb00258.x 1917688

[39] Aghazarian A Huf W Pflüger H Klatte T. Standard semen parameters vs. sperm kinematics to predict sperm DNA damage. World J Mens Health (2021) 39(1):116–22. doi: 10.5534/wjmh.190095 PMC7752507 31749338

[40] Pregl Breznik B Kovačič B Vlaisavljević V. Are sperm DNA fragmentation, hyperactivation, and hyaluronan-binding ability predictive for fertilization and embryo development in *In vitro* fertilization and intracytoplasmic sperm injection? Fertil Steril (2013) 99(5):1233–41. doi: 10.1016/j.fertnstert.2012.11.048 23290739

[41] Burkman LJ. Characterization of hyperactivated motility by human spermatozoa during capacitation: comparison of fertile and oligozoospermic sperm populations. Arch Androl (1984) 13(2-3):153–65. doi: 10.3109/01485018408987514 6537743

[42] Kay VJ Robertson L. Hyperactivated motility of human spermatozoa: a review of physiological function and application in assisted reproduction. Hum Reprod Update (1998) 4(6):776–86. doi: 10.1093/humupd/4.6.776 10098469

[43] Fénichel P Donzeau M Farahifar D Basteris B Ayraud N Hsi BL. Dynamics of human sperm acrosome reaction: relation with in vitro fertilization. Fertil Steril (1991) 55(5):994–9. doi: 10.1016/s0015-0282(16)54312-x 2022277

[44] Salmon-Divon M Shrem G Balayla J Nehushtan T Volodarsky-Perel A Steiner N. An age-based sperm nomogram: the mcgill reference guide. Hum Reprod (2020) 35(10):2213–25. doi: 10.1093/humrep/deaa196 32914183

[45] Bahadur G Almossawi O Zeirideen Zaid R Ilahibuccus A Al-Habib A Muneer A. Semen characteristics in consecutive ejaculates with short abstinence in subfertile males. Reprod BioMed Online (2016) 32(3):323–8. doi: 10.1016/j.rbmo.2015.11.021 26776821

[46] Blackwell JM Zaneveld LJ. Effect of abstinence on sperm acrosin, hypoosmotic swelling, and other semen variables. Fertil Steril (1992) 58(4):798–802. doi: 10.1016/s0015-0282(16)55330-8 1426327

[47] Sokol P Drakopoulos P Polyzos NP. The effect of ejaculatory abstinence interval on sperm parameters and clinical outcome of art. a systematic review of the literature. J Clin Med (2021) 10(15):3213. doi: 10.3390/jcm10153213 34361997 PMC8347289

[48] Keihani S Craig JR Zhang C Presson AP Myers JB Brant WO. Impacts of abstinence time on semen parameters in a Large population-based cohort of subfertile men. Urology (2017) 108:90–5. doi: 10.1016/j.urology.2017.06.045 28712886

[49] Agarwal A Gupta S Du Plessis S Sharma R Esteves SC Cirenza C. Abstinence time and its impact on basic and advanced semen parameters. Urology (2016) 94:102–10. doi: 10.1016/j.urology.2016.03.059 27196032

[50] Alipour H van der Horst G Christiansen OB Dardmeh F Jørgensen N Nielsen HI. Improved sperm kinematics in semen samples collected after 2 h versus 4-7 days of ejaculation abstinence. Hum Reprod (2017) 32(7):1364–72. doi: 10.1093/humrep/dex101 28531319

[51] Barbagallo F Cannarella R Crafa A Manna C La Vignera S Condorelli RA. The impact of a very short abstinence period on conventional sperm parameters and sperm DNA fragmentation: a systematic review and meta-analysis. J Clin Med (2022) 11(24):7303. doi: 10.3390/jcm11247303 36555920 PMC9782170

[52] Sauer MV Zeffer KB Buster JE Sokol RZ. Effect of abstinence on sperm motility in normal men. Am J Obstet Gynecol (1988) 158(3 Pt 1):604–7. doi: 10.1016/0002-9378(88)90038-5 3348323

[53] Levitas E Lunenfeld E Weiss N Friger M Har-Vardi I Koifman A. Relationship between the duration of sexual abstinence and semen quality: analysis of 9,489 semen samples. Fertil Steril (2005) 83(6):1680–6. doi: 10.1016/j.fertnstert.2004.12.045 15950636

[54] Marshburn PB Giddings A Causby S Matthews ML Usadi RS Steuerwald N. Influence of ejaculatory abstinence on seminal total antioxidant capacity and sperm membrane lipid peroxidation. Fertil Steril (2014) 102(3):705–10. doi: 10.1016/j.fertnstert.2014.05.039 24993799

[55] Agarwal A Saleh RA Bedaiwy MA. Role of reactive oxygen species in the pathophysiology of human reproduction. Fertil Steril (2003) 79(4):829–43. doi: 10.1016/s0015-0282(02)04948-8 12749418

[56] Gosálvez J González-Martínez M López-Fernández C Fernández JL Sánchez-Martín P. Shorter abstinence decreases sperm deoxyribonucleic acid fragmentation in ejaculate. Fertil Steril (2011) 96(5):1083–6. doi: 10.1016/j.fertnstert.2011.08.027 21924714

[57] Pons I Cercas R Villas C Braña C Fernández-Shaw S. One abstinence day decreases sperm DNA fragmentation in 90 % of selected patients. J Assist Reprod Genet (2013) 30(9):1211–8. doi: 10.1007/s10815-013-0089-8 PMC3800522 23996278

[58] Aitken RJ Irvine DS Wu FC. Prospective analysis of sperm-oocyte fusion and reactive oxygen species generation as criteria for the diagnosis of infertility. Am J Obstet Gynecol (1991) 164(2):542–51. doi: 10.1016/s0002-9378(11)80017-7 1992700

[59] Alkan I Simşek F Haklar G Kervancioğlu E Ozveri H Yalçin S. Reactive oxygen species production by the spermatozoa of patients with idiopathic infertility: relationship to seminal plasma antioxidants. J Urol (1997) 157(1):140–3. 8976236

[60] Sharma RK Agarwal A. Role of reactive oxygen species in Male infertility. Urology (1996) 48(6):835–50. doi: 10.1016/s0090-4295(96)00313-5 8973665

[61] Agarwal A Sharma RK Sharma R Assidi M Abuzenadah AM Alshahrani S. Characterizing semen parameters and their association with reactive oxygen species in infertile men. Reprod Biol Endocrinol (2014) 12:33. doi: 10.1186/1477-7827-12-33 24885775 PMC4047553

[62] Ko EY Sabanegh ES Jr. Agarwal A. Male Infertility testing: reactive oxygen species and antioxidant capacity. Fertil Steril (2014) 102(6):1518–27. doi: 10.1016/j.fertnstert.2014.10.020 25458618

[63] Guz J Gackowski D Foksinski M Rozalski R Zarakowska E Siomek A. Comparison of oxidative Stress/DNA damage in semen and blood of fertile and infertile men. PloS One (2013) 8(7):e68490. doi: 10.1371/journal.pone.0068490 23874641 PMC3709910

[64] Aitken RJ Clarkson JS Fishel S. Generation of reactive oxygen species, lipid peroxidation, and human sperm function. Biol Reprod (1989) 41(1):183–97. doi: 10.1095/biolreprod41.1.183 2553141

[65] Iwasaki A Gagnon C. Formation of reactive oxygen species in spermatozoa of infertile patients. Fertil Steril (1992) 57(2):409–16. doi: 10.1016/s0015-0282(16)54855-9 1735495

[66] Okada FK Andretta RR Spaine DM. One day is better than four days of ejaculatory abstinence for sperm function. Reprod Fertil (2020) 1(1):1–10. doi: 10.1530/raf-20-0018 PMC8812405 35128419

[67] Peralta-Arias RD Vívenes CY Camejo MI Piñero S Proverbio T Martínez E. Atpases, ion exchangers and human sperm motility. Reproduction (2015) 149(5):475–84. doi: 10.1530/rep-14-0471 25820902

[68] Tvrdá E Ďuračka M Benko F Kováčik A Lovíšek D Gálová E. Ejaculatory abstinence affects the sperm quality in normozoospermic men-how does the seminal bacteriome respond? Int J Mol Sci (2023) 24(4):3503. doi: 10.3390/ijms24043503 36834909 PMC9963725

[69] Pellestor F Girardet A Andreo B. Effect of long abstinence periods on human sperm quality. Int J Fertil Menopausal Stud (1994) 39(5):278–82. 7820161

[70] Liu MM Liu L Chen L Yin XJ Liu H Zhang YH. Sleep deprivation and late bedtime impair sperm health through increasing antisperm antibody production: a prospective study of 981 healthy men. Med Sci Monit (2017) 23:1842–8. doi: 10.12659/msm.900101 PMC5402839 28412762

[71] Li X Wang X Wu Q Guo R Leng X Du Q. Short total sleep duration and poor sleep quality might be associated with asthenozoospermia risk: a case-control study. Front Physiol (2022) 13:959009. doi: 10.3389/fphys.2022.959009 36277203 PMC9581216

[72] Nordkap L Priskorn L Bräuner EV Marie Hansen Å Kirstine Bang A Holmboe SA. Impact of psychological stress measured in three different scales on testis function: a cross-sectional study of 1362 young men. Andrology (2020) 8(6):1674–86. doi: 10.1111/andr.12835 32621382

[73] Janevic T Kahn LG Landsbergis P Cirillo PM Cohn BA Liu X. Effects of work and life stress on semen quality. Fertil Steril (2014) 102(2):530–8. doi: 10.1016/j.fertnstert.2014.04.021 PMC4382866 24856463

[74] Gollenberg AL Liu F Brazil C Drobnis EZ Guzick D Overstreet JW. Semen quality in fertile men in relation to psychosocial stress. Fertil Steril (2010) 93(4):1104–11. doi: 10.1016/j.fertnstert.2008.12.018 19243749

[75] Zang J Yu H Zhu Z Lu Y Liu C Yao C. Does the dietary pattern of shanghai residents change across seasons and area of residence: assessing dietary quality using the chinese diet balance index (Dbi). Nutrients (2017) 9(3):251. doi: 10.3390/nu9030251 28282864 PMC5372914

[76] Salas-Huetos A Bulló M Salas-Salvadó J. Dietary patterns, foods and nutrients in Male fertility parameters and fecundability: a systematic review of observational studies. Hum Reprod Update (2017) 23(4):371–89. doi: 10.1093/humupd/dmx006 28333357

[77] Safarinejad MR. Effect of omega-3 polyunsaturated fatty acid supplementation on semen profile and enzymatic anti-oxidant capacity of seminal plasma in infertile men with idiopathic oligoasthenoteratospermia: a double-blind, placebo-controlled, randomised study. Andrologia (2011) 43(1):38–47. doi: 10.1111/j.1439-0272.2009.01013.x 21219381

[78] Nassan FL Chavarro JE Tanrikut C. Diet and men's fertility: does diet affect sperm quality? Fertil Steril (2018) 110(4):570–7. doi: 10.1016/j.fertnstert.2018.05.025 30196939

[79] Eslamian G Amirjannati N Rashidkhani B Sadeghi MR Hekmatdoost A. Intake of food groups and idiopathic asthenozoospermia: a case-control study. Hum Reprod (2012) 27(11):3328–36. doi: 10.1093/humrep/des311 22940769

[80] Salas-Huetos A Maghsoumi-Norouzabad L James ER Carrell DT Aston KI Jenkins TG. Male Adiposity, sperm parameters and reproductive hormones: an updated systematic review and collaborative meta-analysis. Obes Rev (2021) 22(1):e13082. doi: 10.1111/obr.13082 32705766

[81] Ramaraju GA Teppala S Prathigudupu K Kalagara M Thota S Kota M. Association between obesity and sperm quality. Andrologia (2018) 50(3). doi: 10.1111/and.12888 28929508

[82] Zhao Y Zhu Q Lin J Cai J. Association of exposure to particulate matter air pollution with semen quality among men in China. JAMA Netw Open (2022) 5(2):e2148684. doi: 10.1001/jamanetworkopen.2021.48684 35175344 PMC8855237

