# Supplementary material for: Trends in sperm quality by computer-assisted sperm analysis of 49,189 men during 2015–2021 in a fertility center from China
Source: Front Endocrinol (Lausanne). 2023 Jul 12;14:1194455. doi: 10.3389/fendo.2023.1194455 (PMC10390301; doi:10.3389/fendo.2023.1194455)
Supplement: Supplementary file 1 [file Table_1.docx]

Supplementary Material

**Trends in Sperm Quality of by Computer-Assisted Sperm Analysis of 49 189 Males During 2015–2021 in China**

**Yanquan Li^1,2*^, Tingting Lu^1,2*^, Zhengmu Wu^1^, Zhengquan Wang^1^, Ting Yu^1,2^, Hanshu Wang^1,2^, Chunhua Tang^1,2#^, Yuchuan Zhou^1,2#^.**

^1^International Peace Maternity and Child Health Hospital, School of Medicine, Shanghai Jiao Tong University, Shanghai, China.

^2^Shanghai Key Laboratory of Embryo Original Diseases, Shanghai, China.

^*^These authors made equal contributions to this work.

**^#^****Correspondence:** Yuchuan Zhou [zhouych@sibcb.ac.cn](mailto:zhouyuchuan2017@163.com); Chunhua Tang [tangchunhua@sibcb.ac.cn](mailto:tangchunhua@sibcb.ac.cn)

**Supplementary Figure**

Line charts showing parameters in the different age and abstinence time groups were shown in Figures S1, S2.

Semen volume (Figure S1A), total motility (Figure S1D), progressive motility (Figure S1E), VCL (Figure S1F), VSL (Figure S1G), and VAP (Figure S1H) showed decreasing trends, and sperm concentration (Figure S1C), BCF (Figure S1I) showed a slightly increasing trend with age. Sperm count (Figure S1B) first rose, then fell.

Semen volume (Figure S2A), sperm count (Figure S2B) and concentration(Figure S2C) showed increasing trends with abstinence time, and total motility (Figure S2D), progressive motility (Figure S2E), VCL (Figure S2F), VSL (Figure S2G), and VAP (Figure S2H) showed decreasing trends with abstinence time especially in BRL group.


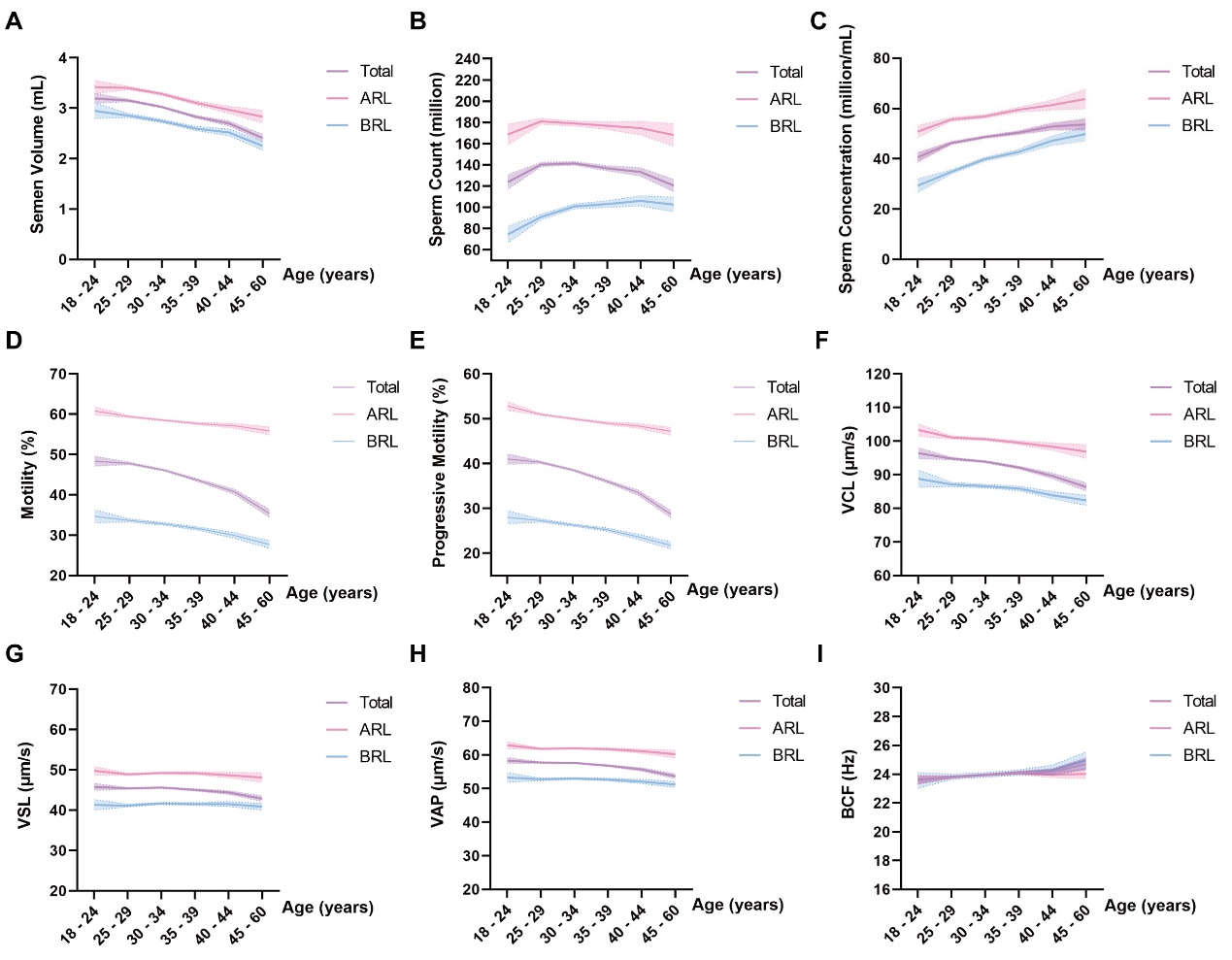


**Supplementary Figure 1**: The line charts of semen analysis by age groups. Means and 95% confidence intervals of semen volume (Figure S1A), sperm count (Figure S1B), sperm concentration (Figure S1C), total motility (Figure S1D), progressive motility (Figure S1E), VCL (Figure S1F), VSL (Figure S1G), and VAP (Figure S1H), BCF (Figure S1I) in groups by age. The purple line represents the total sample, the pink line represents the ARL group, and the blue line represents the BRL group.


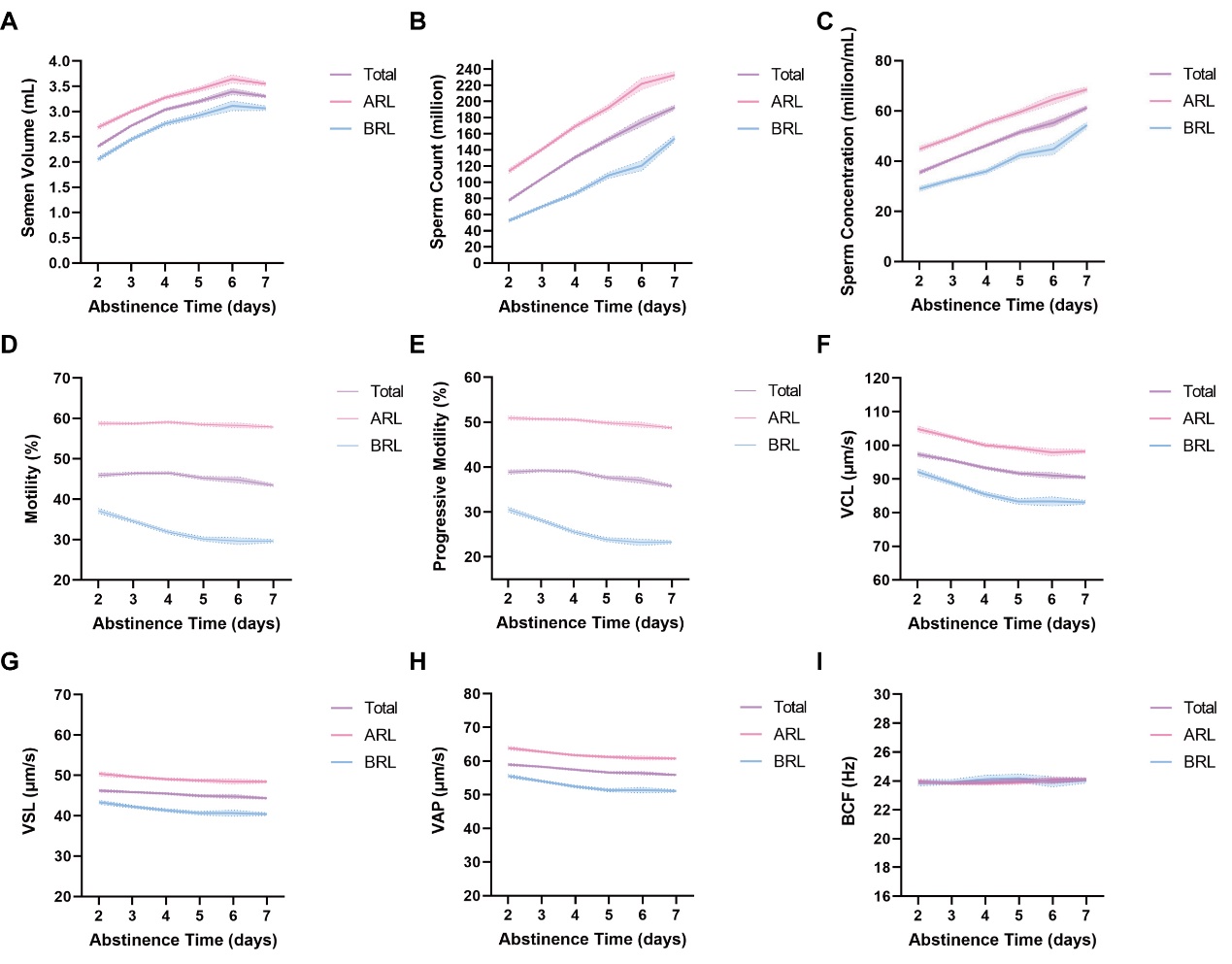


**Supplementary Figure 2**: The line charts of semen analysis by abstinence time groups. Means and 95% confidence intervals of semen volume (Figure S2A), sperm count (Figure S2B), sperm concentration (Figure S2C), total motility (Figure S2D), progressive motility (Figure S2E), VCL (Figure S2F), VSL (Figure S2G), and VAP (Figure S2H), BCF (Figure S2I) in groups by abstinence time. The purple line represents the total sample, the pink line represents the ARL group, and the blue line represents the BRL group.
